# New genus and species of broad-nosed weevils from Baltic amber and notes on fossils of the subfamily Entiminae (Coleoptera, Curculionidae)

**DOI:** 10.3897/zookeys.160.2108

**Published:** 2011-12-29

**Authors:** Nikolai N. Yunakov, Alexander G. Kirejtshuk

**Affiliations:** 1 Natural History Museum, University of Oslo, P.O. Box 1172 Blindern NO-0318 Oslo, Norway; 2 Zoological Institute, Russian Academy of Sciences, Universitetskaya nab. 1, St. Petersburg, 199034, Russia; 3 Muséum National d’Histoire Naturelle, CP 50, Entomologie, 45 Rue Buffon, F- 75005 Paris, France

**Keywords:** new genus and species, new synonymy, fossil Entiminae, Naupactini, Baltic amber, key, checklist

## Abstract

*Arostropsis groehni* **gen. et sp. n.** is described from Baltic amber and temporarily placed in the tribe Naupactini. It differs from all recent Naupactini genera with open corbels by very short and flattened scape, distinct lateral carina of the pronotum and elytra, and the rostrum distinctly narrower than the head capsule. The shape of head in the extinct genus is somewhat similar to that of the extant Naupactini genera with enclosed corbels (*Platyomus* Sahlberg, 1823 and *Aptolemus* Schoenherr, 1842), but differs in the slender body, open corbels, very short antennal scape and epifrons without a median sulcus (only a longitudinal depression is slightly visible). It is also similar to the Tanymecine genus *Pandeleteius* Schoenherr, 1834 in general appearance, but distinct by the straight anterior edge of the pronotum, lack of postocular spurs, lobes, and vibrissae, a slightly sloping elytral declivity, lateral ridges on the pronotum, subflattened antennal scape, elongate rostrum, and sparsely setose epistome. A new synonymy of the generic names *Protonaupactus* Zherikhin, 1971 and *Sucinophyllobius* Wanat & Borowiec, 1986, **syn. n.**, is established. The Madagascan genus *Corecaulus* Fairmaire, 1903 is transferred from the tribe Naupactini to the Brachyderini because of its connate claws and the similarity in chaetotaxy of the epistomal area with African and Madagascar Brachyderini genera. A key to the identification of known Baltic amber genera of Entiminae is proposed. A checklist of the prepleistocene fossil Entiminae, based on V.V. Zherikhin’s data, with remarks and corrections, is presented.

## Introduction

Having examined the Baltic amber weevils from the collection of Mr. Carsten Gröhn, a new genus and species belonging to the subfamily Entiminae is described. However its tribal attribution was questioned because of the aberrant shape of the head capsule and inaccessibility of structures normally used for diagnostics (e.g. genitalia). In studying the various characters of Entiminae weevils, it became readily apparent that one of the most useful diagnostic features at the suprageneric level is the structure of the mandibular processes. They show some variability within Entiminae in general, but within certain tribes is usually rather stable. Besides, the mandibular process of fossils can be used, if available, as an additional character for identification of tribes.

In the course of the current study, mandibular processes of 35 extant genera in 17 tribes, including 6 genera of the tribe Naupactini Gistel, 1848, were examined (results of this comparison will be published in a forthcoming paper).

In addition, non-American genera treated in the Naupactini were also examined. As a result, the Madagascar genus *Corecaulus* Fairmaire, 1903 (type, female examined, MNHN) was transferred from the tribe Naupactini to Brachyderini, as its claws are connate in the basal third and similar to those in the genera *Podionops* Schoenherr, 1847 (South Africa) and *Lagocaulus* Fairmaire, 1903 (Madagascar) with the long median sulcus at the vertex and chaetotaxy of the epistomal area.

The type specimens of some poorly known taxa were re-examined. *Phyllobius sobrinus* Voss, 1972 was placed in the genus *Sucinophyllobius* (Wanat and Borovec 1986). A redescription of *Protonaupactus* is provided and a new taxonomic placement is proposed for this genus in accordance with the current knowledge of the Tertiary Entiminae fauna (Zherikhin 1971, 1992; and original data). This new information is included in the online catalogue (Ponomarenko et al. 2011).

## Historical review of fossil Entiminae

The earliest descriptions of fossils from the subfamily Entiminae Schoenherr, 1823 were published by Heer (1847), Germar (1849), and Giebel (1856) in their reviews of “Tertiary” insects. One of the preceding publications includes a brief note on unrecognizable “*Naupactus*” species (Serres 1829). These authors assigned fossil species to recent genera. The oldest species treated as Entiminae (*Sitonites melanarius*) was dated from the Upper Jurassic (Heer 1864), but this assignment with the broad-nosed weevils is rather doubtful and it is not considered reliable. The fossil specimens reliably identified as Entiminae originated from the Middle Eocene (Green River and Roan Mountain), the boundary between the Eocene and Oligocene, and also from the Lower Oligocene (Florissant and White River) (Scudder 1893). Exclusively rich American deposits with 47 genera and European deposits with 11 genera demonstrate the level of the Caenozoic diversity of this subfamily. The Middle Eocene deposits contain taxa resembling Entiminae but bear no mandibular processes, such as in Aterpini Lacordaire, 1866, usually assigned to the subfamily Cyclominae Schoenherr, 1826 (Oberprieler 2010). Some advanced genera of Entiminae have also been found with well developed postocular vibrissae and mandibles bearing mandibular processes, which are usually treated as members of the tribes Tanymecini Lacordaire, 1863 and Ophryastini Lacordaire, 1863. A rather large number of the taxa with free claws in the Caenozoic fossil weevils could be regarded as remarkable. Unfortunately, poor preservation of weevils from the Green River and most other American sediments make it difficult to determine generic or even tribal attributions, as most important characters (particularly legs and thin structures of the rostrum) seem to be rarely available for study. On the other hand, good preservation of some compression remnants makes it possible to provide precise systematic interpretations. In particular, the inprints of *Geralophus fossicius* Scudder, 1893 from Florissant, initially treated as Alophini LeConte, 1874, have the distinct transverse sulcus at the base of the epifrons, and the latter was transferred to Cylydrorhinini Lacordaire, 1863 or Aterpini (Cyclominae) (see Scudder 1893: plate II, Figs 16, 17 and 24); however, a more precise placement is scarcely possible due to the masking of fine structures of the mouthparts and epistomal area of the rostrum. Most Entiminae described from the Upper Eocene resin of Denmark, Poland and Russia (Voss 1953, 1972; Zherikhin 1971; Wanat and Boroviec 1986) are in quite good condition.

## Baltic amber Entiminae and their systematic and biographic links

The Baltic amber weevils apparently share more similarity with recent groups occurring mostly in the Indo-Malayan (Oriental) and Neotropical Regions (Zherikhin 1971). The biotic similarity could be due to the more homogeneous Paleogene Euro-Asian biota which now mostly remains in the recent “Himalayan-Burmanian-Yunannian block” (Takhtajan 1970; Kirejtshuk and Kurochkin 2010), but the link between this Paleogene biota with the recent Neotropical fauna is still unclear. Nevertheless, the Baltic amber fauna of Entiminae comprises two fossil genera, *Paonaupactus* Voss, 1953 and *Protonaupactus* Zherikhin, 1971, linked to the Neotropics, versus *Sucinophyllobius* Wanat & Boroviec, 1986, linked to the Indo-Malayan Region. *Polydrusus* Germar, 1817, is the only confirmed recent genus known from Baltic amber. It was established by the paleoendemic, monotypic subgenus *Palaeodrosus* Zherikhin, 1971, and is highly diverse in the recent fauna of the warm-temperate zone of the Palaearctic where it comprises 204 species. Another recent Palaearctic genus recorded from Baltic amber may be *Trachyphloeus* Germar, 1824 (Klebs 1910), but this data is still not confirmed.

The genus *Paonaupactus* Voss, 1953 is monotypic and it is considered as a member of the tribe Anypotactini Champion, 1911 (Alonso-Zarazaga and Lyal 1999). *Protonaupactus* Zherikhin, 1971 is also monotypic and it was originally placed in the tribe Naupactini Gistel, 1856). The genus *Sucinophyllobius* Wanat & Boroviec, 1986 was proposed for *Phyllobius sobrinus* Voss, 1972 and another species (*Sucinophyllobius viridis* Wanat & Boroviec, 1986) was also described in it. According to the recent catalogue of weevil genera (Alonso-Zarazaga and Lyal 1999), *Sucinophyllobius* belongs to the tribe Cyphicerini Lacordaire, 1863. Preliminary study and a comparison of *Sucinophylobius* with *Cyphicerus* reveals that the characters of the head and prothorax make it questionable if *Sucinophyllobius* belongs within the subtribe Cyphicerina (see further discussion below under *Protonaupactus*).

## Methods

The usual optic equipment was used for descriptions, including a Leica MZ 16.0 microscope provided with a CCD camera and camera lucida. Morphological terms mostly follow Thompson (1992), Emden (1944), and Doyen (1966). Special terms related to the rostrum structure follow Oberprieler 1988 and details of the epistomal area follow Morimoto et al. (2006).

*Measurements.* All measurements were taken with an ocular-micrometer. Body length was measured from the anterior margin of the eyes to the apex of the elytra, and rostrum length from the rostrum apex to the anterior margin of the eyes. Width of the rostrum is the maximum distance between the lateral edges of the pterygia.

*Imaging.* All outline illustrations were drawn using a camera-lucida and modified with a Wacom Graphire 4 Classic XL A5 tablet in Corel Draw (version 11.633) Corel®. Merging of layers was done with Helicon Focus (version 5.0) HeliconSoft®. Amber samples were photographed as under normal conditions as well as in sugar syrup to provide more suitable light refraction.

*Abbreviations of depositories*. **GPIH** Institute of Geology and Palaeontology and Museum (Geologo-Paläontologisches Institut u. Museum), University of Hamburg; **MNHN** National Museum of Natural History (Museum National d’Histoire Naturelle), Paris; **ZMUC** Zoological Museum (Zoologisk Museum), University of Copenhagen.

*Abbreviations of morphological terms*. **cb** corbel, **es** epistomal setae, **ed** elytral declivity, **fr** frons, **hp** humeral prominence, **ibt** intero-basal tooth, **ma** mandibular process, **lr** lateral ridge of pronotum, **pep** parepistome.

*Abbreviations in table.* **Agri** Agriento (=Girgenti), Sicilia, Italy, Upper Miocene; **Aix** Aix-en-Province, France, Lower Oligocene; **BalJ** Baltic Amber, Baltic and North Sea coast, Upper Eocene; **Boet** Böttinger Marmors, Germany, Miocene; **Cere** Céreste, west to Apt, Alpes-de-Haute, Basses Alp Department, Provence, France, Lower Oligocene; **Cela** Célas, railway Uzés - Saint-Julien-de-Casignac, Fumades, Corents, Bassein Ales, Gard Dept., France, Upper Eocene, previously Lower Oligocene (Sannosien); **Dece** Lava Camp Mine, Jumachuk River Valley, Seward peninsula, Alaska, Pleocene-Pleistocene (5.7 mln - 27 000 - Deceit Formation); **DomJ** Dominican amber, Dominican Republic and Haiti; Lower Miocene; **CerG** Cerro Guido, Ultima Esperanza, Magallanes, Upper Cretaceous; **Core** Corent, Gergovia Plateu, south of Clermon-Ferran, Puy-de-Dom Department, France, Upper Oligocene; **Flor** Florissant, south fork of Twin Creek, Front Range near Pike’s Peak, Colorado, U.S.A., Lower Oligocene. **GreR** Green-River, 3-4 km western rail-way crossing of Green River; Utah, U.S.A, Middle Eocene; **N1** Neogene, Miocene; **N11** Neogene, Lower Miocene; **N13** Neogene, Upper Miocene; **N2** Neogene, Pliocene; **K2** Upper Cretaceous; **Dece** Lava Camp Mine, Jumachuk River Valley, Seward peninsula, Alaska, Pleocene-Pleistocene site (5.7 mln - 27 000 - Deceit Formation); **Oeni** Oeningen, near Baden lake, Baden-Württemberg, Germany, Upper Miocene; **Pg12** Paleogene, Middle Paleocene; **Pg2** Paleogene, Eocene; **Pg22** Paleogene, Middle Eocene; **Pg23** Paleogene, Upper Eocene; **Pg31** Paleogene, Lower Oligocene; **Pg33** Paleogene, Upper Oligocene; **RoaM** Roan Mountain, Colorado, USA, Middle Eocene; **Rott** Siebengebirge, Germany; Lower Miocene, Aquitanian or Upper Oligocene; **Sunc** Sunchal, La Mendieta, Jujuy Prov., northern Argentina; Upper Paleocene (Lower Eocene); **WhiR** White River Badlands, South Dakota, boundary Eocene and Oligocene.

## Taxonomic treatment

**Order Coleoptera** Linnaeus, 1758

**Family Curculionidae** Latreille, 1802

**Subfamily Entiminae** Schoenherr, 1823

### Arostropsis

Yunakov & Kirejtshuk gen. n.

urn:lsid:zoobank.org:act:24BF76B1-8ADA-44BA-B111-FEED72EB7A05

http://species-id.net/wiki/Arostropsis

#### Type species.

*Arostropsis groehni* Yunakov & Kirejtshuk, sp. n.

#### Etymology.

 The name of the new genus is formed from the Greek negative prefix “a”, “rōstron” (beak, bill, snout) and “opsis” (resembling a (specified) thing). Gender feminine.

#### Diagnosis.

 Body elongate, in general appearance similar to *Pandeleteius* Schoenherr, 1834. Antenna with scape short, as long as pedicel (first funicular article; term after Doyen 1966) and 2^nd^ funicular article (antennomere 3) together, reaching anterior margin of eye, strongly widened and flattened dorso-ventrally along apical third. Rostrum distinctly elongate and slender, about half as thick as frons. Vertex with small frontal fossa anterior of eyes. Buccal cavity completely covered by prementum; transverse sulcus absent. Epistome sparsely setose: only two pairs of short (internal) and long (external) setae (such composition of setae is not found among groups of Naupactini and Anypotactini); parepistome (term after Morimoto et al. 2006) weakly acute and gently extending beyond contour of rostrum (Fig. 14). Antennal scrobes entirely lateral, just swinging fossa (term after Morimoto et al. 2006) slightly visible from above, pterygia scarcely pronounced. Mandibular process resembling that in other Naupactini (this structure was examined for six genera of Naupactini), weakly curved inward, with distinct dorsal carina, without inner basal tooth (Fig. 18). Eyes strongly asymmetrically convex. Pronotal and elytral disk distinctly depressed dorsally. Pronotum with distinct lateral ridges; its posterior edge bisinuate. Elytra subparallel-sided, with humeral and distinct subapical prominences, elytral declivity gently sloping. Femora obtuse, weakly swollen. Metatibiae with open corbels. Claws free.

#### Arostropsis groehni

Yunakov & Kirejtshuk sp. n.

urn:lsid:zoobank.org:act:25FBCC0C-8376-41C7-A86E-F024B1541EEC

http://species-id.net/wiki/Arostropsis_groehni

**Figures 1–6.**
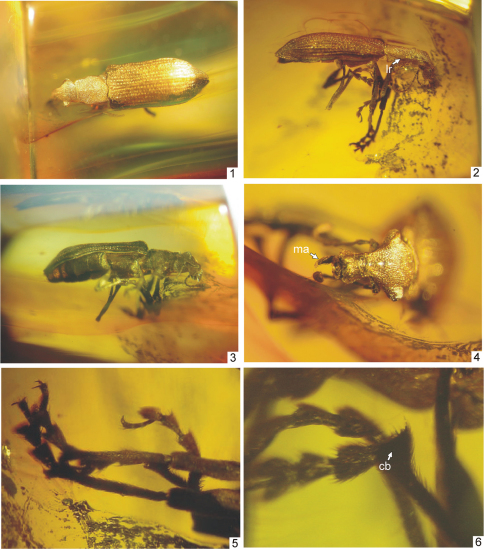
*Arostropsis groehni* gen. et sp.n. **1** body, dorsal view **2** body, lateral view **3** body, ventro-lateral view **4** head, dorsal view **5** protarsi **6** metatibia apex. Abbreviations: **cb** – corbel, **ma** – mandibular process. Body length: 6.4 mm.

Figs 1 [Fig F2] [Fig F3] –16

##### Material examined.

*Holotype* “C 7968, GPIH 4516”, male (GPIH); the complete beetle with a clear integument is included in an irregular parallelepiped with the largest plane about 18.0 × 14.0 mm and the smallest one 11.0 × 7.0 mm; amber matter on right side from the beetle is rather homogeneous, but that from the left side of the inclusion consists of some layers, in which between the borders is a fine net of dark (almost blackish) organic matter.

##### Etymology.

 The epithet of the new species is formed from the name of Carsten Gröhn, collector of its holotype.

##### Type strata.

 Baltic Amber; Upper Eocene, Prussian Formation.

##### Type locality.

 Baltic Sea coast and Amber quarry Jantarny near Kaliningrad (formerly Koenigsberg), Kaliningrad region, Russia.

##### Description.

 Length 6.4, width 1.8, height 1.3 mm. Body slender, distinctly depressed from above. Pronotum and elytra strongly carinate at sides. Integument densely covered with small, apparently metallic, lanceolate (apparently green) scales at both sides of body and legs.

*Head*. Rostrum 1.5 times as long as wide, narrow, distinctly narrower than head capsule, laterally depressed. Pterygia weakly extending beyond lateral contour of rostrum. Epistomal area not depressed. Lateral edges of epifrons at middle convex, narrowed towards middle, then parallel-sided, without basal, transverse depression or sulcus. Median sulcus shallow, extending towards pit between eyes, not continuing towards vertex. Head capsule not constricted beyond eyes. Frons distinctly convex, almost twice as wide as epifrons at level of antennal insertions. Maximum width of head at posterior part of eyes. Scape comparatively short, about 1/4 as long as funicle, strongly expanded apically, somewhat compressed dorsoventrally, not extending beyond anterior third of eyes, directed obliquely downwards in folded state. Funicle slender; all segments elongate, funicular segment 1 about three times as long as wide, 2^nd^ about two times as long as wide, 3^rd^ about 1.5 times as long as wide; 4^th^-5^th^ about two times as long as wide, segments 6-7 about 1.5 times as long as wide. Club spindle-shaped and comparatively thin, with distinctly separated segments, about as long as funicular segments 1-7 together. Mandibles entirely bare (without scales), moderately extended beyond buccal cavity. The only remaining left mandibular process knife-shaped, in apical third slightly curved inward, without internal prominences, about 1.5 times as long as protruding part of mandibles.

*Pronotum* elongate, almost parallel-sided, with anterior and posterior constrictions widely expressed, weakly and evenly convex at sides, with disc weakly convex transversely and anterior edge curving upward; posterior edge slightly bisinuate; posterior angles widely rounded and somewhat projecting posteriorly; anterior edge nearly straight.

**Figures 7–8. F2:**
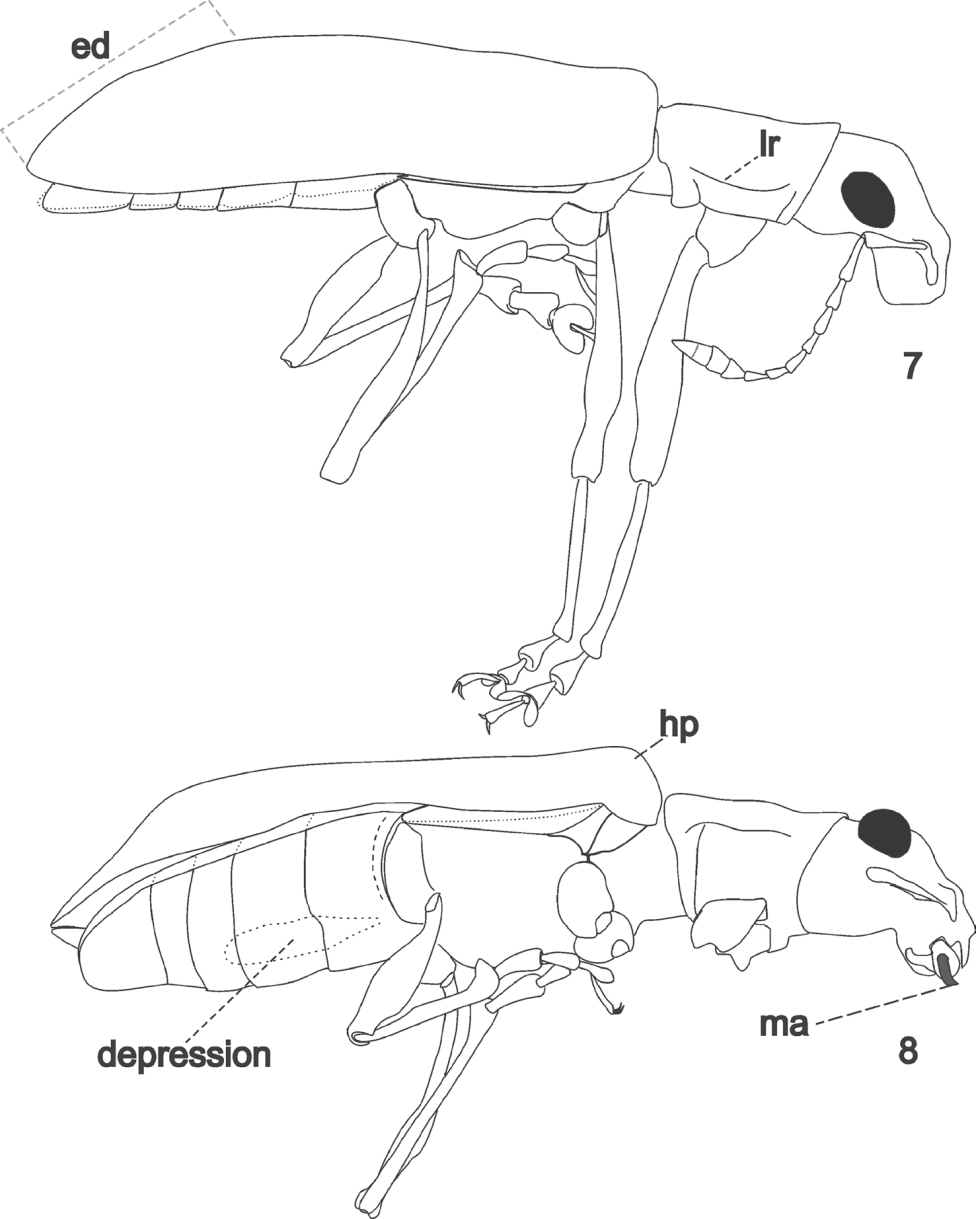
*Arostropsis groehni* gen. et sp.n. **7** body outline, lateral view **8** idem, ventro-lateral view (anterior and middle limbs removed). Abbreviations: **ed** elytral declivity, **hp** humeral prominence, **lr** lateral ridge, **ma** mandibular process. Body length: 6.4 mm.

*Elytra* elongate, about 4.5 times as long as wide, parallel-sided, humeral prominences distinct (Fig. 8, **hp**), basal edge of elytra biconvex opposite the posterior pronotal depressions. Elytral declivity gently sloping (Fig. 7).

*Legs* slender. Femora slender, elongate, obtuse, weakly swollen in apical third. Tibiae slender and elongate. Protibiae gently curved inwards in apical third; with anterior row of thin spines. Metatibiae subflattened and with inner edge sinuate at apical third. Corbels open (Figs 6, **cb**; 8), without additional row of spines. Tarsi slender, setaceous, pelma well-developed (term after Doyen 1966). Tarsomere 1 almost as long as tarsomeres 2–3 combined. Ultimate tarsomere extended beyond lobes of tarsomere 3 by length of last one. Claws free. Procoxae comparatively small, situated in middle of prothorax (Fig. 8).

**Figures 9–12. F3:**
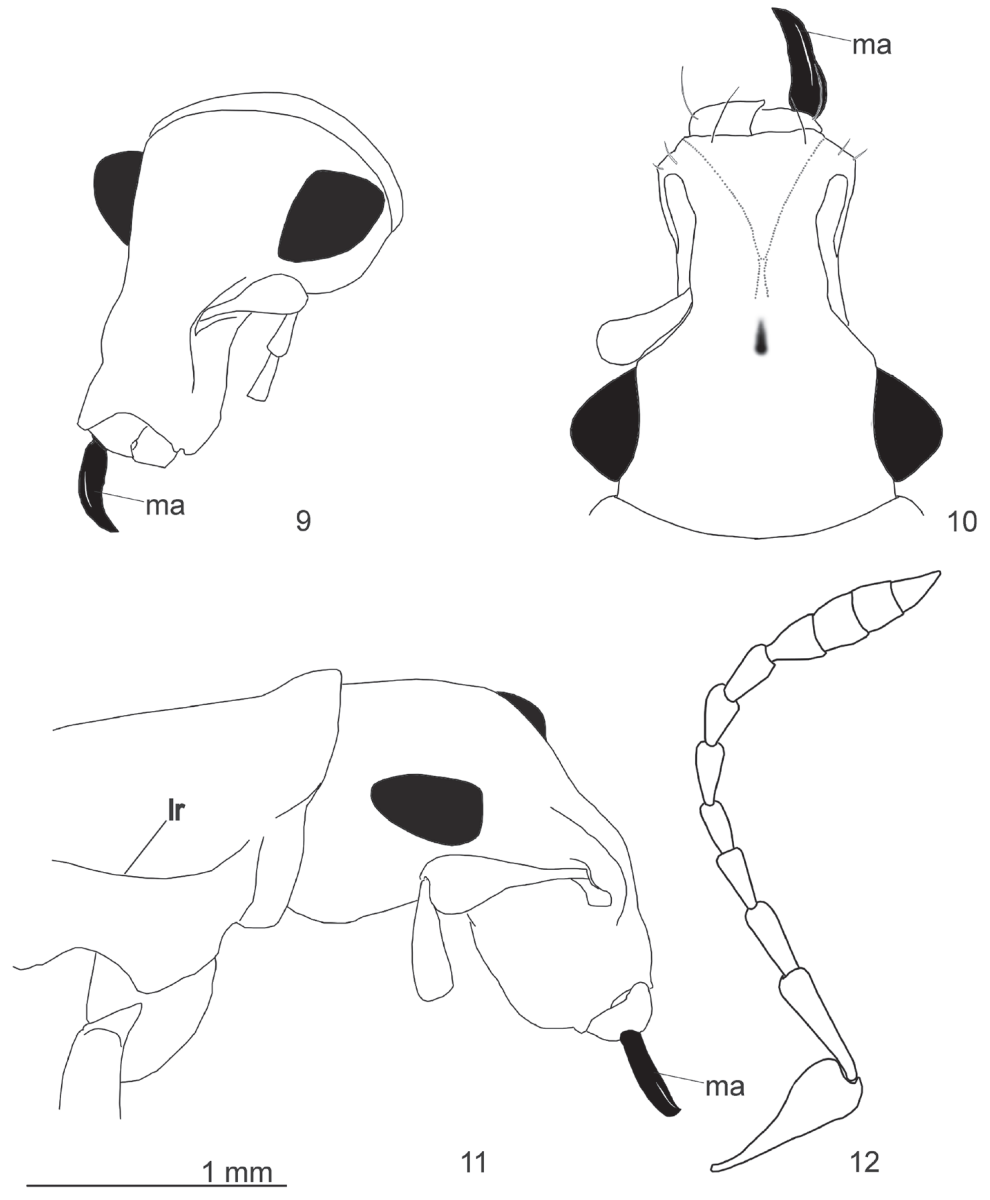
*Arostropsis groehni* gen. et sp.n. **9** head, antero-lateral view **10** idem, antero-dorsal view **11** head and prothorax, lateral view **12** antenna, lateral view. Abbreviations: **lr** lateral ridge, **ma** mandibular process. Scale bar: 1 mm.

**Figures 13–18.**
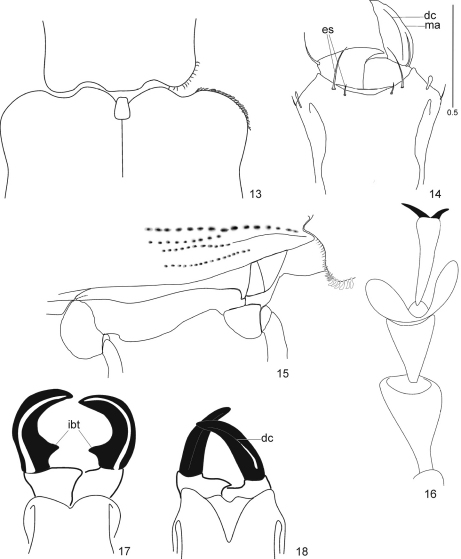
Species of*Arostropsis*, *Lepropus*, and *Naupactus*, details. Figs 17 and 18 (modified from Thompson 1992). **13**–**16**. *Arostropsis groehni* sp. n. **13** base of pronotum and elytra **14** epistome **15** epipleural margin of elytra **16** protarsus **17** *Lepropus rutilans* (Olivier, 1807) (Tanymecini), mandibular processes dentate **18** *Naupactus fatuus* Boheman, 1833, mandibular processes simple (Naupactini). Abbreviations: **dc** dorsal carina of mandibular process, **ibt** interobasal tooth of mandibular process, **es** epistomal setae, **ma** mandibular process. Scale bar: 1 mm (Figs **13, 15**); 0.5 mm (Figs **14, 16**); not in scale (Figs **17, 18**).

##### Comparison with recent genera.

 The new genus differs from all recent genera of Naupactini with open corbels and procoxae not completely separated from the prosternum (*Mesagroicus* Schoenherr, 1840, *Eurymetopus* Schoenherr, 1840, *Melanocyphus* Jekel, 1875, *Trichonaupactus* Hustache, 1939 ) in the following characters: short and depressed scape, rostrum narrow, epistomal area weakly setose, epifrons with a weakly developed median depression and vertex with very small fossa, not continuing to occiput.

##### Comparison with Baltic amber entimine genera.

 Since *Arostropsis* gen. n. has free claws, only two other fossil genera, *Paonaupactus* Voss, 1953 and *Protonaupactus* Zherikhin, 1971, share the same character state and can be compared with the new genus. *Arostropsis* gen. n. strongly differs from both *Paonaupactus* and *Protonaupactus* in the following characters given in the key below.

###### Key to Baltic amber genera of Entiminae with free claws

**Table:** 

1	Scape short, reaching middle of eyes, strongly thickened apically; pedicel 1.5 times as long as or 2^nd^ funicular article; 1^st^ article of club similar in shape and size with 7^th^ funicular article. Eyes irregularly convex, lateral, located significantly below level of frons (in lateral view). Epifrons without transverse sulcus before eyes. Lateral carina of prothorax developed. Pronotal disc depressed. Procoxae situated in middle of prosternum. Body length 6.4 mm	*Arostropsis* gen. n.
–	Scape long, reaching anterior edge of prothorax, not strongly thickened at apex; pedicel 0.7 time as long as 2^nd^ funicular article; 1^st^ article of club significantly different in shape from 7^th^ funicular article. Eyes evenly convex, dorso-laterally, located almost at level of frons and somewhat extended above level of frons (in lateral view). Epifrons with more or less developed, transverse sulcus before eyes. Sides of prothorax evenly swollen, without carina. Pronotal disc moderately convex. Procoxae are closer to the anterior than to the posterior edge of prosternum. Body length 4.0-4.8 mm	2
2	Antennal club oval. 4.5-4.8 mm.	*Protonaupactus* Zherikhin, 1971
–	Antennal club spindle-shaped. 4 mm.	*Paonaupactus* Voss, 1953

###### Systematic position

This new genus is undoubtedly a member of Entiminae due to the presence of mandibular processes. Due to structural characters: mandibular processes long, knife-shaped (**ibt** not developed) - claws free, vertex and epifrons combined in uniform structure without transverse sulcus before eyes, the new genus could be assigned to the tribes Naupactini or Geonemini Gistel, 1848. Emden separated these groups from other ‘brachyderoid’ tribes of Entiminae with free claws (Tanymecini and Anypotactini) by the following characters (Table 1).

**Table 1.** Basic morphological characters of ‘brachyderoid’ tribes of Entiminae with free claws in comparison with the genus *Arostropsis* gen.n.

	Geonemini	Naupactini	Tanymecini	Anypotactini	Arostropsis
1. Postocular vibrissae	absent	absent	present	absent	absent
2. Claws	free	free	free/connate	free	free
3. Transverse sulcus	absent	present/absent	present/<br/> absent	present/<br/> obsolete	absent
4. Eyes position	dorso-lateral	lateral	dorso-lateral	dorso-lateral	lateral
5. Mentum covers maxillae	yes	yes	yes	yes/no	unknown
6. Metatibial corbels	open/enclosed	open/enclosed	open/enclosed	open	open
7. Mandibular processes	without ibt	without ibt	with ibt	unknown	without ibt
8. Procoxae position	anterior	anterior/median	median	median	median

The position of *Arostropsis* is tested following the table:

Presumption 1 (Geonemini). *Arostropsis* gen.n. lacks the key characters of Geonemini such as: evenly sloping lateral edges of epifrons, very narrow vertex and anterior position of procoxae. Consequently this genus cannot be assigned to this tribe..

Presumption 2 (Tanymecini). *Arostropsis* gen. n. could not be considered in the tribe Tanymecini, due to absence of postocular vibrissae at the prothorax. The amount of vibrissae varies from genus to genus within Tanymecini but at least a few vibrissae are present (some *Pandeleteius*). We do not know any case in which vibrissae are completely absent, so it is unlikely that *Arostropsis* belongs to Tanymecini.

Presumption 3 (Anypotactini). Due to absence of transverse sulcus between vertex and epifrons and U-shaped epistome in *Arostropsis* it is impossible to consider this genus within Anypotactini.

Presumption 4 (Naupactini). The strictly lateral position of the eyes, flattened epifrons and very broad vertex resembles that of *Arostropsis* within Naupactini, however, the shape of the rostrum is very different from that of any known member of this tribe. This transformation of rostrum probably resulted in reduction of the frontal fossa (Fig. 10) that is normally (in genera with broad rostrum) deep, long and continuing from the anterior portion of epifrons to the occiput. Such head shape together with the unusual slender body makes it difficult to recognize *Arostropsis* as a member of the tribe Naupactini.

**Probable bionomy**. The long legs and well developed tarsal pelma (term after Doyen 1966) of the new species suggests that this beetle was capable of running swiftly between leaves and, therefore, it was likely a canopy-dweller.

### Genus Protonaupactus

Zherikhin, 1971

Sucinophyllobius Wanat & Borowiec, 1986: 243, syn. n. Type species *Sucinophyllobius viridis* Wanat & Boroviec, 1986

#### Type species.

*Protonaupactus microphthalmus* Zherikhin, 1971

#### Included species:

*Protonaupactus microphthalmus* Zherikhin, 1971, *Protonaupactus viridis* (Wanat & Boroviec, 1986), comb. n., *Protonaupactus sobrinus* (Voss, 1972), comb. n.

#### Protonaupactus sobrinus

(Voss, 1972) comb. n.

http://species-id.net/wiki/Protonaupactus_sobrinus

**Figures 19–24.**
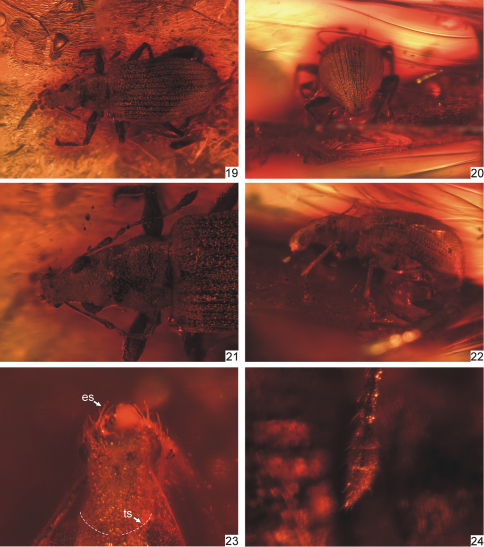
*Protonaupactus sobrinus* (Voss, 1972), holotype, male. **19** body, dorsal view **20** elytral declivity, posterior view **21** head and pronotum, dorsal view **22** body, lateral view **23** rostrum, dorsal view (transverse sulcus, indicated by dashed line, hidden by dense scale vestiture) **24** antennal club. Abbreviations: **es** epistomal setae, **ts** transverse sulcus. Body length: 4.5 mm.

Figs 19 [Fig F6] –34

Phyllobius sobrinus Voss 1972: 174
Sucinophyllobius sobrinus Wanat & Borowiec 1986: 243

##### Material examined.

**Holotype:** ZMUC903847, male (ZMUC); the complete beetle is included in a nearly regular amber piece, parallelepiped, with facets 14.0, 10.0 and 5.0 mm; one thin layer with wavy, light organic matter, small gas bubbles and small cracks located beneath the beetle.

##### Type strata.

 Baltic Amber; Upper Eocene, Prussian Formation.

##### Type locality.

 Denmark “Bernschteinschluss, Versterhavet bei Thisted, 17.xii.1895, Madsen leg.”.

##### Redescription.

 Length 4.5, width 1.75, and height 1.65 mm. Beetle densely covered with metallic, shining scales (apparently green).

*Head*. Rostrum 1.5 times as long as wide at level of pterygia. Epifrons subparallel-sided, at level of antennal insertions abruptly widened anteriorly, weakly convex longitudinally, separated from frons by a distinct depression indicating a transverse sulcus hidden by dense scales. Pterygia strongly extending beyond lateral contour of rostrum. Epistome well-defined by U-shaped keel. Epistomal setae grouped in dense bunches at anterior epistomal angles. Each bunch bearing at least 5 setae. Epistomal angles pronounced apically. Anterior edge of pterygia densely setose. Antennal furrows distinct only in their basal half and not continuing obliquely to underside of rostrum, their dorsal and ventral edges somewhat divergent posteriorly. Eyes dorso-lateral, round and evenly convex. Frons slightly convex, as wide as epifrons between antennal insertions. Frontal fossa not visible because masking by scales. Antennae long. Scape straight, evenly thickened apically, reaching anterior constriction of pronotum. Funicle thin: pedicel about 0.7 time as long as 2^nd^ funicular article; 2^nd^ article about 3.5 times as long as wide; 3^rd^ –7^th^ articles weakly oblong, about 1.5 times as long as wide. Club ovoid, about 2.3 times as long as wide, its 1^st^ article significantly different in shape from 7^th^ funicular article.

*Pronotum* transverse; evenly convex at sides and disc, strongly constricted anteriorly and posteriorly. Its base slightly bisinuate. Anterior edge of pronotum straight, without postocular lobes, spurs or vibrissae. Scutellum significantly pronounced, subquadrate.

*Elytra* subparallel, hardly widened in apical half, with pronounced humeral prominences. Epipleural edge weekly but distinctly S-shaped. Wings well-developed, beetle apparently capable of flying. Elytral declivity abruptly sloping. Anterior edge of anal ventrite (hypopygidium) emarginate (Fig. 30).

*Legs* thin. Femora obtuse, weakly swollen in middle part. Protibiae straight at external edge, not widened at external apical angle. Metatibiae spatulate apically (Fig. 33), with open corbel but without additional setal row. Setal comb of all tibiae weakly-developed. Tarsi with well-developed setaceous pelma (term after Doyen 1966). Tarsomere 1 about as long as tarsomeres 2 and 3 combined. Tarsomere 3 with well-developed lobes. Ultimate tarsomere by 0.7 extending beyond lobes of tarsomere 3. Claws free.

**Figures 25–30. F6:**
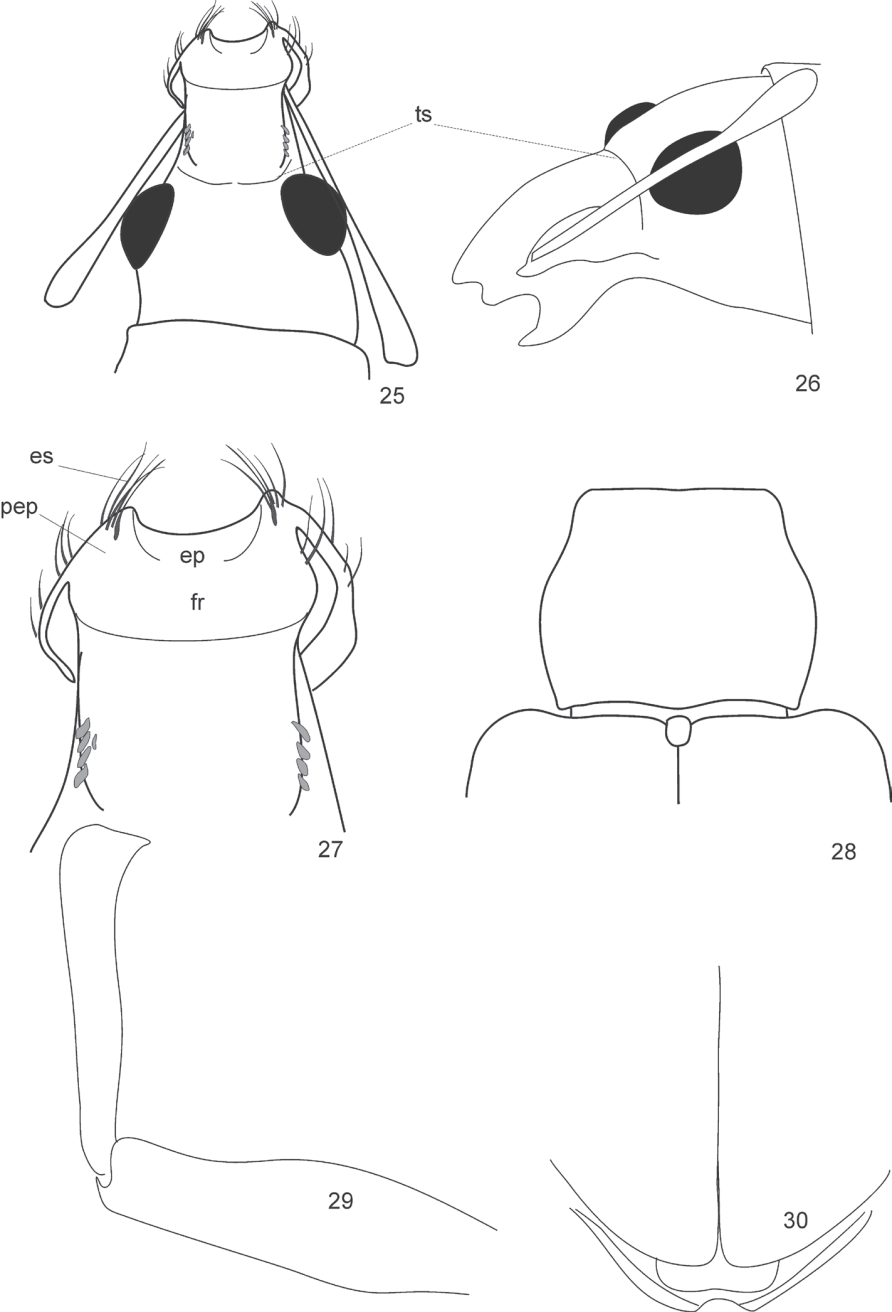
*Protonaupactus sobrinus* (Voss, 1972), holotype, male. **25** head with antennal scape, dorsal view **26** idem, lateral dorsal view **27** epistomal area of rostrum **28** pronotum and elytral base, dorsal view **29** anterior right leg, dorsal view **30** elytral apex with pygidium and anal ventrite. Abbreviations: **ts** transverse sulcus, **es** epistomal serae, **ep** epistome, **fr** frons, **pep** parepistome. Scale bar: 1 mm (Figs 25, 26, 28, 30); 0.5 mm (Figs **27, 29**).

**Figures 31–34.**
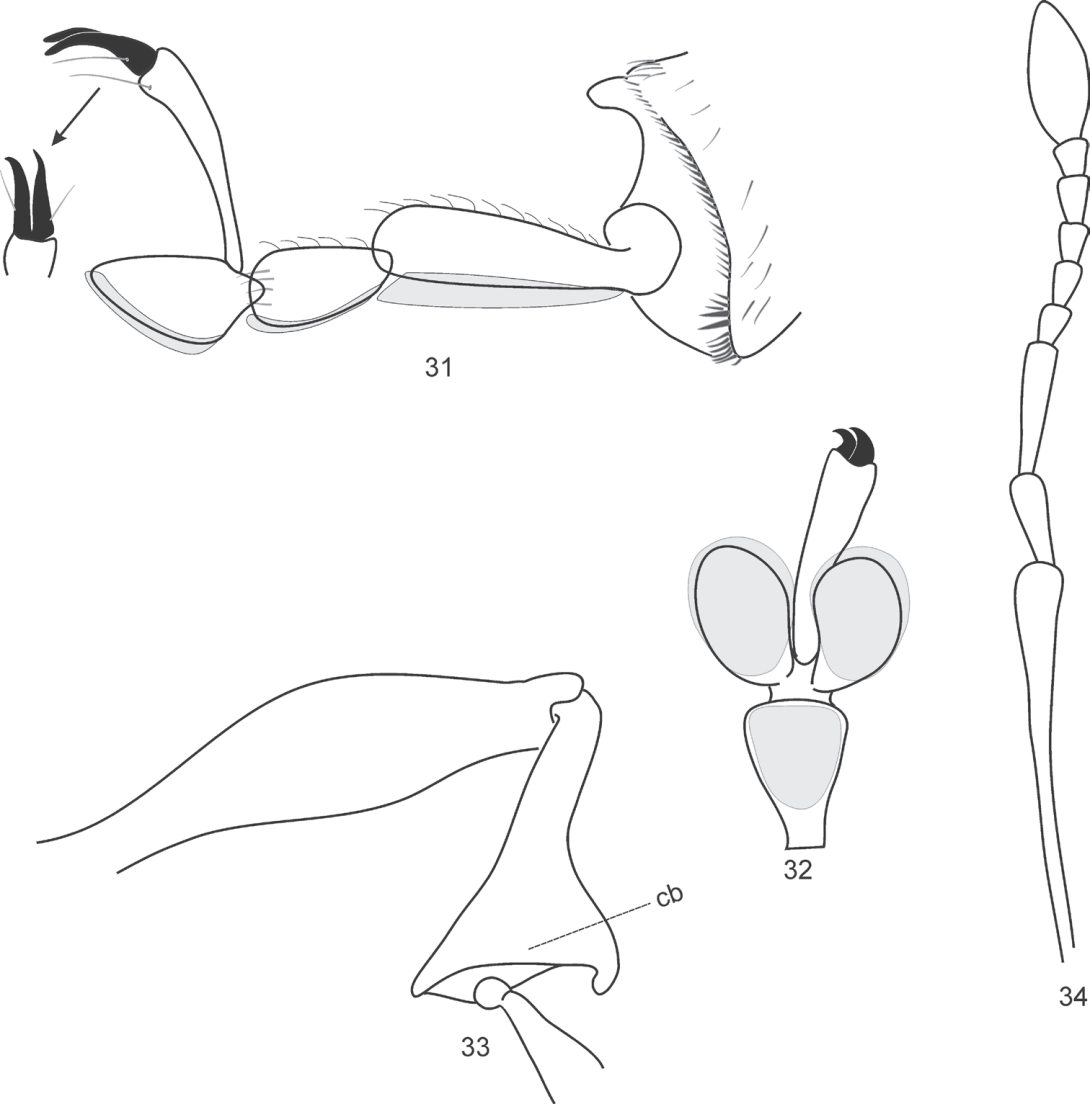
*Protonaupactus sobrinus* (Voss, 1972), holotype, male. **31** metatibial apex and tarsus lateral view, and claws dorsal view **32** mesotarsus ventral view **33** posterior leg **34** antenna. **Remark**. Setose pelma (term after Doyen 1966) indicated by grey color. Scale bar: 0.25 mm (Figs 31, 32); 0.5 mm (Figs **33, 34**).

##### Differential diagnosis.

 This species is distinguished from *Protonaupactus microphthalmus* and *Protonaupactus viridis* by the metatibiae being strongly spatulate apically. The re-examination of the holotype of *Phyllobius sobrinus* Voss, 1972 demonstrates the particular structure of the head, which is very similar to some genera of Anypotactini (mostly from tropical America) and Cyphicerini (mostly from Old World tropics, distinctly separated groups): epistome U-shaped, convex, weakly sinuate anteriorly, and claws free (Figs 27, 31). This species also shares a well-developed, transverse sulcus on the rostrum (Fig. 25, 26, 35–39) with other groups of Anypotactini.

**Figures 35–39.**
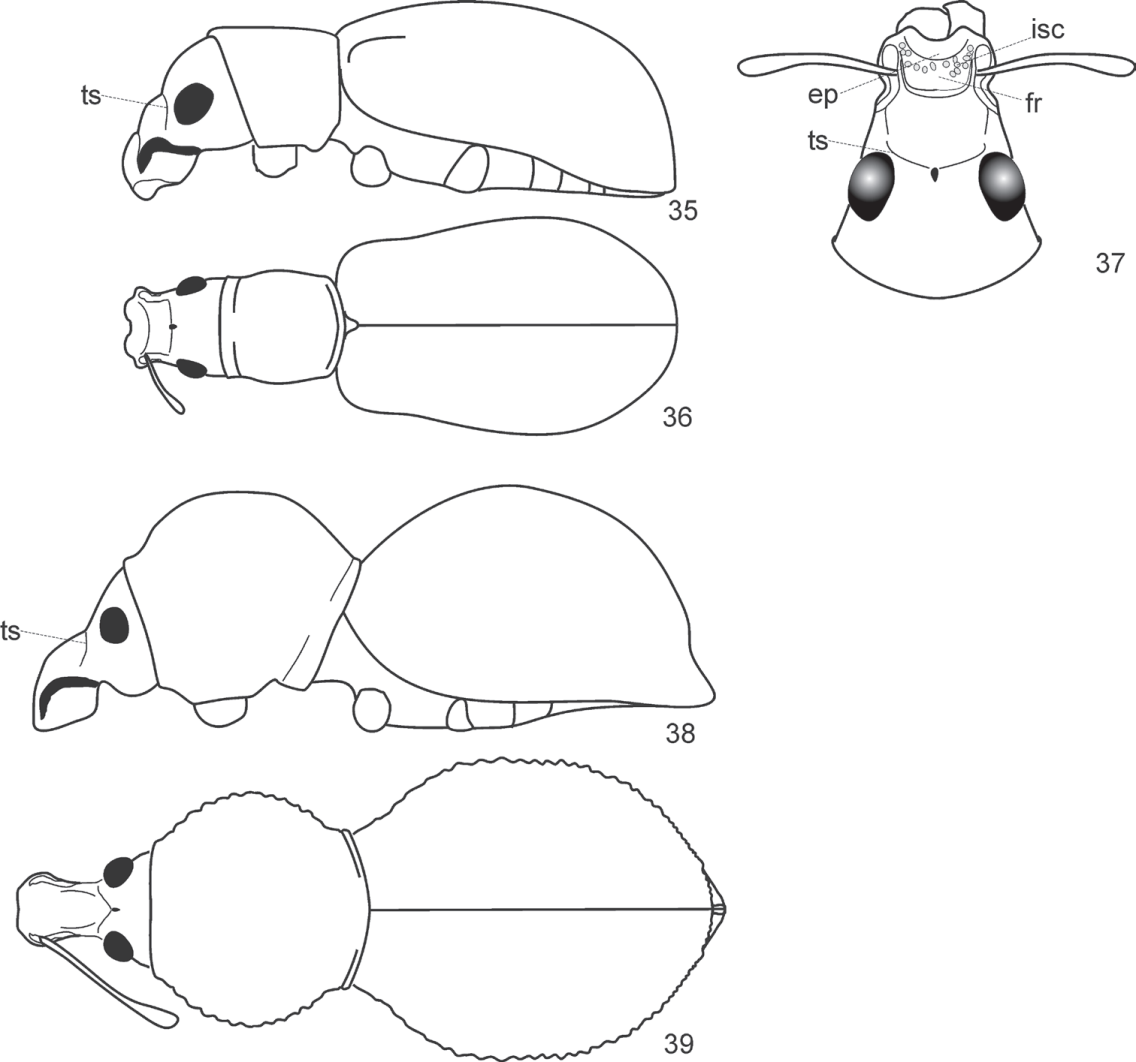
Anypotactini, details. 35, 38 body, lateral view; 36, 39 body, dorsal view; 37 head, dorsal view. **35**–**37** *Anypotactus exilis* Boheman, 1840, female (Venezuela); length 3,3 mm **38**, **39** *Hyphantus buccifer* Germar, 1824, female (Brazil); length 8,2 mm. Abbreviations. **isc** incrustation scales, **ep** epistome, **fr** frons, **ts** transverse sulcus.

##### Notes on synonymy.

 The comparison of *Sucinophyllobius sobrinus*, *Sucinophyllobius viridis* and *Protonaupactus microphthalmus* shows they are closely related species of the same genus. They share almost the same characters of the rostrum, with moderately defined U-shaped epistome having apically pronounced angles, long and slender antennae with the scape extending behind the anterior margin of the pronotum, funicular article 1 about 0.7 × as long as article 2, and article 2 about 3.5 × as long as wide.

###### Systematic position of Protonaupactus

Anypotactini and Cyphicerini are the only tribes of Entiminae whose relationships with *Protonaupactus* still need to be tested. Both tribes contain genera with the following characters: (1) claws free, (2) epistome deeply sinuate, parepistome distinctly protruding and bearing dense bunches of long setae, (3) swinging fossae long, fully visible in dorsal view, (4) pterygia strongly extended beyond rostrum.

The tribe Cyphicerini consists of rather diverse genera which may be divided into two subgroups by the structure of the prothorax and underside of the head: (**1**) **a** prosternum subflattened with straight anterior edge; **b** pronotum with straight anterior edge; **c** rostrum and head united in a consolidated structure; **d** underside of rostrum densely covered with scales, its sculpture uniform; **e** rostrum directed forwards (Mylacorrhinina and Myllocerina) or (**2**) **a** prosternum with anterior edge sinuate; **b** pronotum with distinct postocular lobes; **c** head and rostrum distinctly separated by transverse constriction; **d** basal area of underside of rostrum with glabrous integument which has a bare stripe reaching the eyes; **e** rostrum directed downwards (Acanthotrachelina, Cyphicerina, and Phytoscaphina).

Anypotactini include a group of genera that share a character set characteristic of the subgroup I of Cyphicerini and distinct from this subgroup in the transverse sulcus on the rostrum. However, this tribe displays an enormous variety in rostrum shape, and also shows both dorsal and lateral positions of the antennal scape which is normally folded above the eyes, along the rostrum, or folded obliquely downwards in the scrobe if developed. Both the antennal scape and funicle are quite slender, hence the antennae resemble a brachyderoid rather than otiorhynchoid type, like in Cyphicerini.

*Anypotactus* Schoenherr 1840, *Polydacrys* Schoenherr, 1833 and other genera with short rostrum and large pterygia belong to Anypotactini subgroup 1, and *Prepodellus* Kirsch, 1867, *Hyphantus* Germar, 1824 and other genera with long rostrum with very small pterygia belong to Anypotactini subgroup 2.

*Protonaupactus* combines both character sets, Anypotactini and Cyphicerini. Nevertheless, it can be easily distinguished from Cyphicerini subgr. 2 in sharing the basic character set of Cyphicerini subgr. 1 and Anypotactini subgr. 1. From Cyphicerini subgr.1 *Protonaupactus* differs particularly in the transverse sulcus on the rostrum (Fig. 25, 26). The new data on this fossil genus show that the distinctness between these tribes is not very clear and needs a further investigation.

## Supplementary Material

XML Treatment for Arostropsis

XML Treatment for Protonaupactus

## Appendix

**Checklist of prepleistocene fossil weevils of the subfamily Entiminae**

(according to the taxonomic interpretation in Zherikhin and Egorov 1991 and Ponomarenko et al. 2011)

V.V. Zherikhin analyzed all available sources mentioning fossil Curculionoidea (Jurassic-Paleogene). Later the results of his analysis were published in the catalogue by Ponomarenko et al. (2011). In the list below (Table 1) the authors mostly follow his subdivisions pertaining to the subfamily Entiminae. Many references in the list provided here need to be checked re-examining the fossils. Most accounts regarded as doubtful Zherikhin were removed from the following list. Ponomarenko et al. (2011) treated them as ‘Subfamilia incerta’ or ‘Familia incerta’.

**Table 2.** Checklist of prepleistocene fossil Entiminae. **Subfamilia Entiminae** Schoenherr, 1823

**NN**	**Species**	**age and site of finding**
**Tribe Aterpini** Lacordaire, 1863 (**see note 1 below this table**)		
1	Genus incertus *fossicius* Scudder, 1893:75 (*Geralophus*)	Pg31, Flor
**Tribe Tropiphorini** Marseul, 1863		
2	*Vitavitus thulius* Kissinger, 1973	N2, Dece
3	*Rhytideres sexsulcatus* (Heer, 1856) (*Cleonus*) (= *Hipporhinus reynesii* Oustalet, 1874; *Brachycerus annosus* Oustalet, 1874)	Pg31st, Aix N13, Agri
**Tribe Cylydrorhinini** Lacordaire, 1863		
4	*Dorotheus guidensis* Kuschel, 1959	K2, CerG
**Tribe Sitonini** Gistel, 1848		
5	*Sitona* (subgenus incertus) *lata* Théobald, 1937	?Pg31, unknown
6	*Sitona* (subgenus incertus) sp. (Hieke and Pietrzeniuk 1984)	Pg23, BalJ
**Tribe Entimini** Schoenherr, 1823		
7	*Geralophus antiquarius* Scudder, 1893	Pg31, Flor
8	*Geralophus occultus* (Scudder, 1876) (*Eurhinus*)	Pg31, Flor
9	*Geralophus lassatus* Scudder, 1893	Pg31, Flor
10	*Geralophus scudderi* Wickham, 1911	Pg31, Flor
11	*Eudomus robustus* Scudder, 1893	Pg31, Flor
12	*Eudomus pinguis* Scudder, 1893	Pg31, Flor
13	*Hipporhinops sternbergi* Cockerell, 1926	Pg12, Sunc
14	Genus incertus *saxatilis* Scudder, 1876 (*Eudiagogus*)	Pg22, GreR
15	Genus incertus *effossus* Scudder, 1876 (*Eudiagogus*)	Pg22, GreR
16	Genus incertus *compactus* Scudder, 1878: 765 (*Ophryastes*)	Pg22, GreR
17	Genus incertus *heeri* Germar, 1849 (*Hipporhinus*) (= *matheroni* Nicolas, 1891; *similis* Nicolas, 1891; *intermedius* Nicolas, 1891; *marioni* Nicolas, 1891; *pertonii* Nicolas, 1891 (*Hipporhinus*))	Pg31, Aix
18	Genus incertus *brevis* Giebel, 1856 (*Hipporhinus*)	Pg31, Aix
19	Genus incertus *schaumi* Heer, 1856 (*Hipporhinus*)	Pg31, Aix
20	Genus incertus *pumiceus* Scudder, 1893 (*Geralophus*)	Pg31, Flor
21	Genus incertus *repositus* Scudder, 1893 (*Geralophus*)	Pg31, Flor
22	Genus incertus *atavus* Oustalet, 1870 (*Bagous*)	Pg33, Core
**Tribe Tanymecini** Lacordaire, 1863 (**see note 2 below this table**)		
23	Genus incertus *rugosus* Deichmüller, 1881 (*Thylacites*)	Pg3, Kucl
24	*Protainophthalmus asperulus* (Heer, 1856) (*Cleonus*) (including Théobald, 1937) (= *Brachyderes aquisextanus* Oustalet, 1874, *Brachyderes longipes* Heer in Oustalet, 1874, *Cleonus marcelli* Oustalet, 1874)	Pg31, Aix, Cere
25	*Protainophthalmus margarum* (Germar, 1849) (*Sitona*)<br/> (=*antiqua* Giebel, 1856) (*Sitona*)	Pg31, Aix
26	*Protainophthalmus punctulatus* (Nicolas, 1891) (*Desmidophorus*)	Pg31, Aix
27	*Protainophthalmus regularis* (Nicolas, 1891) (*Hipporhinus*)	Pg31, Aix
28	*Protainophthalmus rugicollis* (Heer, 1847) (*Lixus*)	N13, Oeni
29	*Protainophthalmus thaisi* Nicolas, 1891 (*Desmidophorus*)	Pg31, Aix
30	*Protainophthalmus tuberculatus* Nicolas, 1891 (*Desmidophorus*)	Pg31,Aix
31	*Lepropus* sp. (Zherikhin, 1992)	N13, Agri
32	Genus incertus *nudus* Wickham, 1912 (*Pandeleteius*)	Pg31, Flor
33	Genus incertus *secunda* Wickham, 1912 (*Paussopsis*)	Pg31, Flor
**Tribe Phyllobiini** Schoenherr, 1826		
34	*Phyllobius* sp. (Spahr 1981; Hieke and Pietrzeniuk 1984 etc.)	Pg23, BalJ
**Tribe Polydrusini** Schoenherr, 1823 (**see note 3 below this table**)		
35	*Polydrusus* (*Palaeodrosus*) *archetypus* Zherikhin, 1971	Pg23, BalJ
36	*Polydrusus* (Subgenus incertus) sp. (Burmeister 1832; Hieke and Pietrzeniuk 1984)	Pg23, BalJ
**Tribe Anypotactini** Champion, 1911		
37	*Paonaupactus sitonitoides* Voss, 1953 (*=Polydrusus scheelei* Voss, 1953; *Otiorhynchus pellucidipes* Voss, 1972)	Pg23, BalJ
38	*Paonaupactus cephalotes* (Voss, 1972) (*Phyllobius*)	Pg23, BalJ
39	*Protonaupactus microphthalmus* Zherikhin, 1971	Pg23, BalJ
40	*Protonaupactus sobrinus* (Voss, 1972) (*Phyllobius* sbg. *Subphyllobius*); Wanat et Borowiec, 1986 (*Sucinophyllobius*)	Pg23, BalJ
41	*Protonaupactus viridis* (Wanat et Borowiec, 1986) (*Sucinophyllobius*)	Pg23, BalJ
**Tribe Hormorini** Horn, 1876 (see note 4 below this table)		
42	*Hormorus undulatus* (Uhler, 1855) (*Chlorophanus*) (Alonso-Zarazaga and Lyal 1999)	N1, USA
**Tribe Pristorhynchini** Heer, 1847 (Pristorhynchiden)		
43	*Pristorhynchus ellipticus* Heer, 1847	N13, Oeni
**Tribe Naupactini** Gistel, 1856		
44	Genus incertus *florissantensis* Wickham, 1914 (*Cyphus*)	Pg31, Flor
45	Genus incertus *subterraneus* Wickham, 1911 (*Cyphus*)	Pg31, Flor
**Tribe Otiorhynchini** Schoenherr, 1826		
46	*Otiorhynchus boettingensis* van Emden in Zeuner, 1931	N13, Boet
**Tribe Trachyphloeini** Lacordaire, 1863		
47	*Trachyphloeus* sp. (Klebs, 1910)	Pg23, BalJ
**Tribe Eudiagogini** LeConte, 1874		
48	*Promecops tumidirostris* Poinar & Brown, 2011	N11, DomJ
**Tribe uncertain**		
49	*Laccopygus nilesii* Scudder, 1893	Pg31, Flor
50	*Evopes veneratus* Scudder, 1893	Pg31, Flor
51	*Oligocryptus sectus* (Scudder, 1893) (*Eucryptus*)	Pg31, Flor
52	Genus incertus *atavina* Heer, 1847 (*Sitona*)	N13, Oeni
53	Genus incertus *crassirostris* Heer, 1864 (*Naupactus*)	N13, Oeni
54	Genus incertus *dilapsus* Scudder, 1893 (*Phyxelis*)	Pg22, GreR
55	Genus incertus *durus* Scudder, 1893 (*Cryptorhynchus*)	Pg22, RoaM
56	Genus incertus *eradicatus* Scudder, 1893 (*Phyxelis*)	Pg22, RoaM
57	Genus incertus *evanidus* Scudder, 1893 (*Omileus*)	Pg31, Flor
58	Genus incertus *evigoratus* Scudder, 1893 (*Phyxelis*)	Pg22, WhiR
59	Genus incertus *exanimis* Scudder, 1876 (*Eudiagogus*, *Epicaerus*)	Pg22, GreR
60	Genus incertus *fortis* Cockerell, 1909 (?*Syntomostylus*)	Pg22, GreR
61	Genus incertus *manderstjernai* Heyden et Heyden, 1866 (*Tychius*)	N11, Rott
62	Genus incertus *margarum* Theobald, 1937 (*Sitona*) [HN]	Pg23, Cela
63	Genus incertus *obdurefactus* Scudder, 1893 (*Exomias*)	Pg22, RoaM
64	Genus incertus *occubatus* Scudder, 1893 (*Evopes*)	Pg31, Flor
65	Genus incertus *perditus* Scudder, 1876 (*Otiorhynchus*)	Pg22, GreR
66	Genus incertus *petrarum* Scudder, 1893 (*Ophryastes*)	Pg22, RoaM
67	Genus incertus *recuperatus* Scudder, 1893 (*Lachnopus*)	Pg31, Flor
68	Genus incertus *rhenanus* Meuner, 1923 (*Laccopygus*)	N11, Rott
69	Genus incertus *subteractus* Scudder, 1893 (*Otiorhynchus*)	Pg22, RoaM
70	Genus incertus *subterraneus* Scudder, 1893 (*Scythropus*)	Pg22, GreR, WhiR
71	Genus incertus *terrosus* Scudder, 1878 (*Eudiagogus*)	Pg22, RoaM, WhiR
72	Genus incertus *venustulus* Heyden et Heyden, 1866 (*Sitona*)	N11, Rott
73	Genus incertus sp. (Serres, 1829) (*Naupactus*)	Pg31, Aix

Notes to table 2.

**1**. In contrast to the interpretations of Zherikhin, the tribe Aterpini is traditionally treated as belonging to the subfamily Cyclominae (Alonso-Zarazaga and Lyal 1999, Oberprieler 2010, Bouchard et al. 2011) [in the classification proposed by Zherikhin and Egorov (1991) it is considered in ‘Tropiphorinae’]. Alonso-Zarazaga and Lyal (1999) consider the genus *Hipporhinops* in the subfamily Cyclominae.

**2**. According to V.V. Zherikhin’s unpublished data, genus *Protainophthalmus* Zherikhin, 1992 comprises 7 species originally described within ‘*Cleonus*’, ‘*Sitona*’, ‘*Desmidophorus*’, ‘*Hipporhinus*’ and *‘Lixus’* from the Late Paleogene of France and Germany. *Protainophthalmus asperulus* (Heer, 1856) known by three type prints (see Oustalet 1874: 259–263 + plate 3: Figs 20 and 21), two of them (from Aix) are types. We consider they belong to different species or even different genera: since the first print (see Oustalet 1874: Fig. 20) has a transverse sulcus behind the epifrons, but the second print (Oustalet 1874: Fig. 20) does not have such a transverse sulcus. The original description of *Protainophthalmus* (Zherikhin 1992: 174) also includes two prints (SMF VI 218 and SMF VI 220, Senckenberg Museum) from Agrigento (Italy) which are not types of *Protainophthalmus asperulus*.

**3**. The genus *Phyllobius* Germar, 1824 could not be placed in Polydrusini because of significant differences in head structure: long and closed swinging fossae, antennal scrobes not developed (foveiform) and scape not folding while in rest. V.V. Zherikhin, proposed that the tribes Polydrusini and Phyllobiini are synonyms. He considered that the number of mandibular setae is constant and may be used for discrimination of suprageneric groups (Zherikhin and Egorov 1991). Further study of Polydrusini and Phyllobiini genera showed a great variety of the number of mandibular setae. Recent molecular analysis based on two genes 16S rDNA and 18S rDNA, showed that Polydrusini and Phyllobiini are nested in different branches (Hundsdoerfer et al. 2009).

**4**. According to the current interpretation, the tribe Hormorini is assigned to Entiminae (Alonso-Zarazaga and Lyal 1999). This opinion corresponds to the results of our examination of mandibles, which revealed similarity between *Hormorus* and the North-Pacific genus *Byrsopages* Schoenherr, 1842. The abnormal shape of the mandibles, subflattened dorso-ventrally along the anterior edge and ventral position of the mandibular processes, led Zherikhin to refuse this opinion, and he treated *Hormorus* without subfamily placement as ‘subfamilia incerta’.
